# Is exercise/physical activity effective at reducing symptoms of post-traumatic stress disorder in adults — A systematic review

**DOI:** 10.3389/fpsyg.2022.943479

**Published:** 2022-08-12

**Authors:** Ferozkhan Jadhakhan, Nichola Lambert, Nicola Middlebrook, David W. Evans, Deborah Falla

**Affiliations:** ^1^Centre of Precision Rehabilitation for Spinal Pain (CPR Spine), School of Sport, Exercise and Rehabilitation Sciences, College of Life and Environmental Sciences, University of Birmingham, Birmingham, United Kingdom; ^2^National Institute for Health and Care Research (NIHR) Surgical Reconstruction and Microbiology Research Centre, University of Birmingham, Birmingham, United Kingdom; ^3^Faculty of Health and Education, Manchester Metropolitan University, Manchester, United Kingdom

**Keywords:** post-traumatic stress disorder (PTSD), exercise, effectiveness, trauma, management

## Abstract

**Background:**

Exercise has been used to manage symptoms of post-traumatic stress disorder (PTSD). The effect of exercise on PTSD outcomes has been previously explored in several studies. However, it still remains unclear what type of exercise/physical activity, intensity and duration is most effective for reducing symptoms of PTSD. A systematic review was conducted to determine which forms of exercise or physical activity have the greatest effect on PTSD outcome scores including an evaluation of exercise frequency and volume.

**Methods:**

The following electronic databases were systematically searched from January 1980 to June 2021: MEDLINE, PsycINFO, PubMed and Web of Science. Inclusion criteria were studies investigating adults aged 18 or over, reporting the effect of exercise and physical activities on PTSD symptom outcome scores. Two reviewers independently extracted information on study characteristics, exposure and outcomes. In total of 3,217 articles were screened and 23 full text articles further assessed, with 13 RCT's included in the review, covering seven exercise/physical activity interventions. The study protocol was registered prospectively with PROSPERO (CRD42021255131).

**Results:**

Thirteen studies from four countries involving a total of 531 patients were selected for inclusion. Individual forms of exercise/physical activity examined showed some effect on reducing PTSD symptoms but combined exercises (resistance training, aerobic, strength and yoga) administered over a 12 week period, three times a week for 30–60 min showed greater effects on PTSD symptoms.

**Conclusion:**

The limited evidence suggests that a combined exercise intervention has the best evidence for a having a beneficial effect on PTSD symptoms.

**Systematic review registration:**

https://www.crd.york.ac.uk/prospero/display_record.php?RecordID=255131.

## Introduction

Post-traumatic stress disorder (PTSD) is a mental health disorder that can affect people of any age, either immediately or years after direct or indirect exposure to a traumatic event (American Psychiatric Association., 2013). Such events can range from car accidents, combat situations, physical assaults or sudden bereavement (Williamson et al., 2019). It is estimated that 1 in 3 adults will experience a traumatic event at some point in their lives (McManus et al., 2016). The symptoms of PTSD may include intrusion (e.g., flashbacks), hyper-arousal (e.g., heightened alert and an enhanced startle reflex), avoidance of situations or places that remind them of the traumatic event, emotional detachment from their feelings or others, and negative thoughts about themselves (Bisson et al., 2015). An acute reaction to a traumatic event can resolve within a month, but for those left untreated this may last several years (Kessler et al., 1995; Smid et al., 2012). In some cases PTSD can manifest later in life (late onset), sometime years after the experience of a traumatic event (Chopra, 2018). While the average duration if treated is 36 months, approximately one third may still not fully resolve (Smid et al., 2012).

In a large sample of the general population of England, the 2014 Adult Psychiatric Morbidity Survey (APMS) (Baker, 2020) found that 3.7% of men and 5.1% of women aged 16 and over met criteria for PTSD. The Trauma Screening Questionnaire (TSQ) was used to examine the experience of PTSD with a total of ≥6 points out of a possible 10 indicating a positive screen for PTSD (Brewin et al., 2002). Prevalence rates for PTSD in different countries range from 1 to 12% and this range is thought to be due to a number of factors including study methodologies, diagnostic criteria used, likelihood of experiencing an event, and social factors (Klein and Alexander, 2009). Certain groups such as military personnel and those working in emergency services have a greater incidence of PTSD (Perkonigg et al., 2000; McFarlane et al., 2009; Fear et al., 2010; Greenberg et al., 2011).

Current National Institute for Health and Care Excellence (NICE) guidelines (NICE, 2018) recommend a range of treatments depending on the severity and time from trauma, which varies from ‘watch and wait', Cognitive Behavioral Therapy (CBT), Eye Movement Desensitization and Reprocessing (EMDR) and medication. Previous systematic reviews and meta-analyses found moderate effects of exercise on depression, anxiety and PTSD (Bartley et al., 2013; Jayakody et al., 2014; Mura et al., 2014; Stonerock et al., 2015; Kvam et al., 2016; Ravindran et al., 2016; Schuch et al., 2016; Stubbs et al., 2016, 2017; Asher et al., 2017; Krogh et al., 2017). These results were not sufficiently reliable to ensure stable long term benefit. A recent review confirmed these findings showing a small to medium effect (ES: 0.29, 95% CI: 0.10–0.49, *P* < 0.01) of exercise on PTSD symptom severity whereas a sub-group analysis showed no significant effect of exercise on anxiety and depression severity (Bjorkman and Ekblom, 2021). A previous Cochrane review (Lawrence et al., 2010) attempted to investigate the benefits of exercise in the context of sport and games for PTSD but found only five studies, none of which met the review inclusion criteria, suggesting more research is required to assess the effectiveness of exercise in alleviating symptoms of PTSD. Previous reviews have examined the effect of exercise on PTSD in the general population and found that exercise reduces PTSD symptoms as a standalone intervention or adjunct to usual care; the comparability of the studies included in these reviews were limited by heterogeneity in terms of population characteristics, outcome measures, definitions of comparator group and sampling methods. For example, Rosenbaum et al. (2015a,b) conducted a systematic review to evaluate the effect of physical activity interventions on PTSD. Results from their meta-analysis of four randomized controlled trials (RCTs) with a total of 200 participants revealed that physical activity significantly reduced symptoms of PTSD, compared to control groups (Hedges' g = −0.35, 95% CI [−0.63 to −0.07], *p* = 0.02). The authors, however, rated the quality of the studies as satisfactory, following assessment of allocation concealment, assessor blinding and publication bias.

Another systematic review by Whitworth and Ciccolo (2016) investigated the effects of exercise specifically in veterans with PTSD. This review was based on nine observational, two qualitative and two experimental studies. They concluded that there was some evidence to suggest that exercise could reduce PTSD symptoms. However, the studies included showed heterogeneity in their outcome measures and not all studies were specifically testing the effects of exercise on PTSD. A more recent systematic review by McGranahan and O'Connor (2021) which included four RCTs, focused on exercise training effects on sleep quality and symptoms of anxiety and depression in people with PTSD. They analyzed PTSD scores, but only as a secondary outcome measure. The results suggested a small to moderate effect size (Hedges'd = 0.33, 95% CI [−0.66–0.00], *p* < 0.05) toward improvement in PTSD symptoms. Despite the knowledge gained from this body of work, it still remains unclear what type of exercise and or physical activity (structured or unstructured), and what duration and intensity of exercise is most effective for reducing symptoms of PTSD (Friedman, 2019). Physical activity and/or exercise can be in the form of cycling, walking, household or occupational activity or a moderate intensity exercise such as horse-riding. Structured physical activity is generally planned with an underlying goal. Therefore, the purpose of the current systematic review is to examine the available evidence concerning the effect of various types of physical activity and/or exercise interventions, the frequency of intervention and duration of sessions that has the greatest effect on PTSD symptoms.

## Methods

### Search strategy

A comprehensive search of MEDLINE, PubMed, PsycINFO and Web of Science was conducted for articles published from January 1980 to 25^th^ June 2021. A search strategy was developed using keywords with some modifications to allow for alternative spellings and synonyms (Supplementary material 1). Databases were searched from January 1980 since this was the date PTSD was first classified as a disorder in the Diagnostic and Statistical Manual of Mental Disorders (American Psychiatric Association., 2013). Prior to the search commencing (8^th^ June 2021), the protocol for this review was registered with the International Prospective Register of Systematic Reviews (PROSPERO; identification number CRD42021255131). Following a preliminary search, a pragmatic decision was made to widen the eligibility criteria to include people with PTSD attributed to causes other than only musculoskeletal trauma. The review was conducted and reported in accordance with the Preferred reporting System and Meta-Analysis (PRISMA) 2020 statement (Page et al., 2021) (Supplementary material 2).

### Selection criteria

The selection criteria for inclusion and exclusion of studies were formed following the PICOS (Population, Intervention, Comparator, Outcome and Study design) framework (McKenzie and Brennan, 2021) as follows.

#### Population

Adults (aged ≥18 years) diagnosed with PTSD using a recognized measure of the presence of PTSD, for example, The Diagnostic and Statistical Manual of Mental Disorders (DSM−5 and or DSM-4) (American Psychiatric Association., 2000).

#### Study type

Randomized controlled trials.

#### Intervention

Any form of exercise and/or physical activity interventions, structured or non-structured or a combination of both e.g., resistance training, cardiovascular exercise, walking, occupational activity, Tai-chi, and deep breathing exercise. The term “exercise” is commonly used to describe exercise or physical activity, which is planned, structured and repetitive and which serves to improve physical fitness (Caspersen et al., 1985).

#### Comparator

Any study that compared exercise or physical activity with a control group (e.g., usual care or waiting list) for PTSD.

#### Outcome measures

Any validated measure of PTSD symptoms, either self-report or clinician-rated, using measures such as the Clinician-Administered PTSD Scale (CAPS) (Weathers et al., 2018), the PTSD checklist (PCL-5) (Wortmann et al., 2016) and the Posttraumatic Stress Diagnostic Scale (PDS5) (Foa et al., 2015).

#### Exclusions

Studies not written in English were excluded, as were un-published work in non-peer reviewed journals. Systematic reviews, abstracts and non-experimental study designs were also not considered.

### Study selection

All records retrieved in the database search were imported into the Endnote (Clarivate Analytics, USA) publication management software. Initially, the titles and abstracts were screened, independently by two reviewers (NL and NM), according to the eligibility criteria. The full texts of selected articles were subsequently screened by the same reviewers independently. A screening checklist was used by both reviewers to aid selection. If an article was rated as unsure, it was discussed between the two reviewers. In the event of disagreement between the two reviewers, a third reviewer (DF) adjudicated the eligibility of the article.

### Data extraction

Using a data extraction table, based on guidance from the Cochrane Handbook (Li et al., 2021), two reviewers (NL and NM) independently extracted the following data: study details (author, date, location), sample size, characteristics of the participants (age: mean/range, gender), population description, intervention characteristics (type, duration, frequency, time), comparison group, primary outcome measure (measuring PTSD), final assessment point, statistical methods, results and key findings. Any discrepancies were resolved by discussion and re-visiting the relevant study. A third reviewer (DF) was available to mediate any disagreement in data extraction.

### Risk of bias in individual studies

The internal validity of the randomized controlled trials was assessed using the revised Cochrane risk-of-bias tool for randomized trials (RoB 2) (Sterne et al., 2019). Two reviewers (NL and NM) independently assessed each of the included studies. A third assessor (DF) was available if needed. The RoB 2 tool consists of five domains of bias: bias arising from the randomization process, bias due to deviations from intended interventions, bias due to missing outcome data, bias in the measurement of the outcome, and bias in the selection of the reported result. For the assessment of bias due to deviations from intended interventions, the reviewers aimed to include all reported randomized participants (intention to treat analysis) in the final analysis and measure all participants outcome (effect of assignment) regardless of whether description of contamination between trial arms were reported in the studies. These effects will vary between studies if some patients do not receive the intervention or deviate from the assigned intervention (Schünemann et al., 2021). The reviewers used the RoB 2 Cribsheet to follow the algorithm to come to an assessment of bias for the individual domains and then to make an overall judgement of the study as either low risk, some concerns or a high risk of bias.

### Evaluation of the certainty of evidence

The Grading of Recommendations Assessment, Development and Evaluation (GRADE) framework (Guyatt et al., 2011) was used to assess the overall level of evidence for the outcome of interest in the included studies. Consistent with GRADE, the quality of the summary evidence was assessed as high, moderate, low or very low. For each study, we evaluated, imprecision, inconsistency, indirectness, risk of bias including publication bias. Applicability of results were categorized by the study interventions and rated when making judgement about the quality of evidence presented in the included studies (Guyatt et al., 2011). Two members of the review team (NL and NM) independently conducted the GRADE rating using the guidance presented in the Cochrane Handbook (Schünemann et al., 2021). Any disagreements between reviewers were resolved in consultation with a third reviewer (DF).

### Data analysis

Given the significant clinical and statistical heterogeneity between the studies included in this review, it was not possible to pool effect estimates of physical activity/exercise on PTSD symptoms using meta-analysis. Instead, we summarized effect estimates (mean difference or standardized mean difference) with 95% confidence intervals (CI) where appropriate in the included studies. Standardized Mean Difference (SMD) was extrapolated from reported (Cohen's *d*) values, exploring mean difference between groups; an effect size of 0.8 or greater was considered a relatively large effect size between two means in the sample population. A reporting guideline, synthesis without meta-analysis (SWiM) in systematic reviews (Campbell et al., 2020), was used in conjunction with the Preferred Reporting System and Meta-Analysis (PRISMA) 2020 statement (Page et al., 2021) to describe the study results. The SWiM was utilized as the data was not appropriate for meta-analysis because of high heterogeneity between studies in terms of design and definition of exposure and outcome measures, and therefore the results are described narratively.

### Grouping studies for synthesis

Due to the diversity of the interventions, studies were grouped by the type of exercise intervention (aerobic, multi, resistance, sailing, yoga, mindfulness-based stretching and deep breathing exercise) for synthesis and grading the body of evidence rather than pooling across all exercise types. Vote counting was performed to assess if exercise interventions showed a direction of effect for PTSD outcome scores. Each intervention was classified according to whether it suggested a beneficial direction of effect, no change or detrimental effect. This was visually represented, along with the study sample size, in a direction of effect plot. This method of displaying results for a systematic review is deemed acceptable when meta-analysis is not appropriate (McKenzie and Brennan, 2021).

## Results

### Literature search

**Figure 1:**
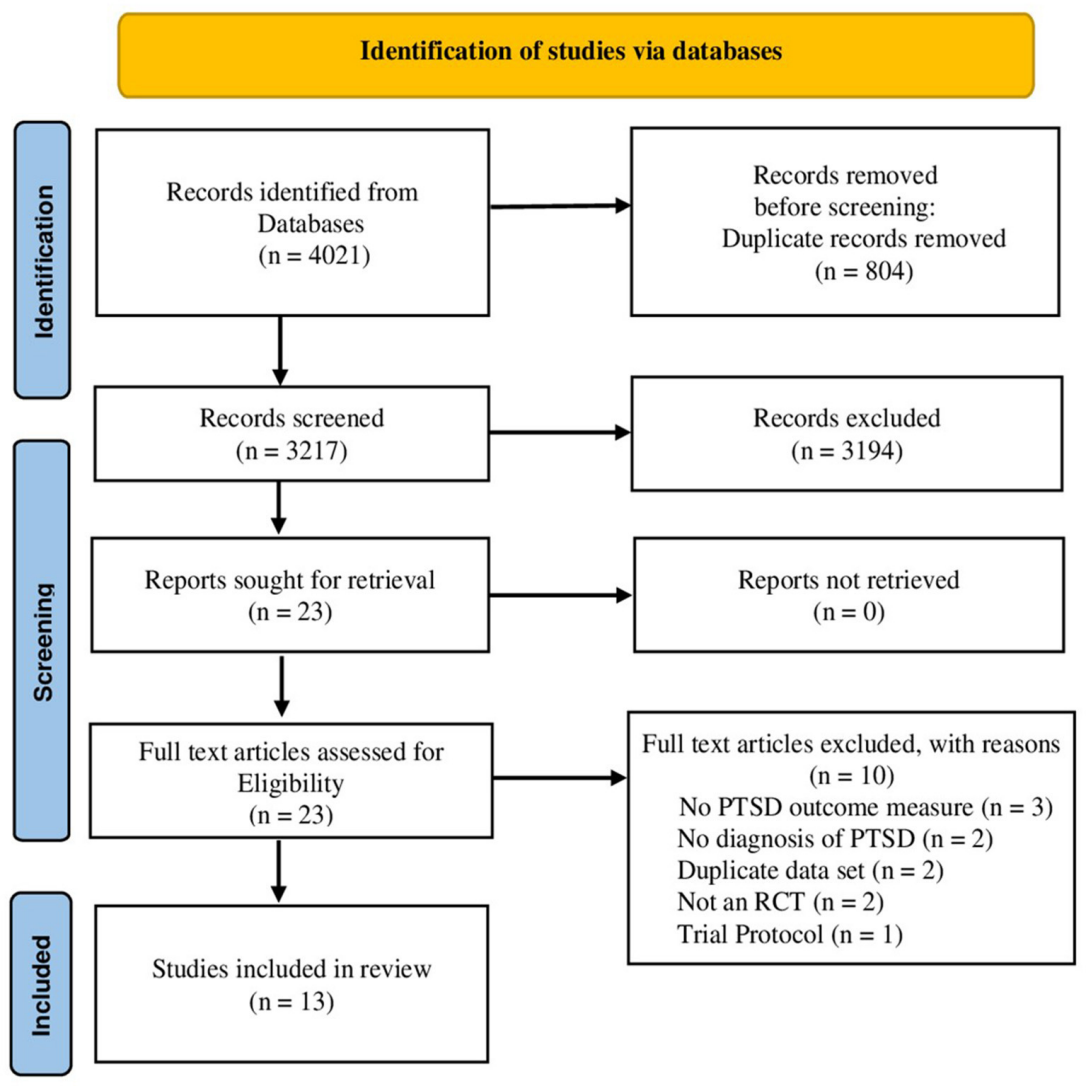
PRISMA Flowchart of the study selection process.

In total, the search strategy yielded 4,021 articles. After excluding 804 duplicates, the titles and abstracts of 3,217 articles were screened for relevance. Title and abstract screening resulted in the exclusion of 3,194 articles, primarily because these articles did not provide data on PTSD outcome, a clear diagnosis of PTSD, or were not a controlled trial. Of the 23 full-text articles that were assessed, 10 were excluded after further review. Three studies were excluded because no PTSD outcome measure was recorded, another two did not report a PTSD diagnosis, a further two studies reported duplicate data. One study was not an RCT and another one was a trial protocol. Thirteen articles were included in the final analysis. A flow diagram of the study selection process is presented in Figure 1.

### Study characteristics

The 13 selected studies (Gelkopf et al., 2013; Kim et al., 2013; Mitchell et al., 2014; van der Kolk et al., 2014; Fetzner and Asmundson, 2015; Powers et al., 2015; Rosenbaum et al., 2015a,b; Goldstein et al., 2018; Johnson et al., 2018; Reinhardt et al., 2018; Whitworth et al., 2019a,b; Hall et al., 2020) involved 531 participants. A significant level of heterogeneity was detected between studies regarding study design including study setting, data source and collection, classification and measures used to define PTSD symptoms, and reported effect estimates. Most studies were performed in the United States (*n* = 10), followed by Canada (*n* = 1), Australia (*n* = 1) and Israel (*n* = 1). Most studies were conducted in a community setting or utilized community care data (*n* = 10), followed by secondary care (*n* = 2) and an Army rehabilitation unit (*n* = 1). Studies included in this review were mostly randomized controlled trials (*n* = 7), followed by pilot RCTs (*n* = 5), and one feasibility/acceptability RCT (*n* = 1). Across all the studies, 54% of participants were male and 46% were female. Males constituted 64% of the intervention group and 34% of comparators. Participants were slightly older in the comparator group (mean age 45.9 years [SD +/−15.1]) compared to the intervention group (mean age 46.9 years [SD+/−15.4]). Race and ethnicity were poorly recorded (number of studies, % of sample): White in intervention group (8, 48.7%) compared to control (6, 22.4%), African-American in intervention group (2, 37.1%) compared to (2, 23.5%) in control group. An overview of the study characteristics is presented in Table 1.

**Table 1:** Description of included studies.

				**Intervention**			**Control**		
**Author and year**	**Location**	**Sample size**	**Setting**	**Age Mean (SD)/ Median [IQR]**	**Gender- *n* (%)**	**Ethnicity- *n* (%)**	**Age- Mean (SD)/ Median [IQR]**	**Gender- *n* (%)**	**Ethnicity – *n* (%)**
Fetzner and Asmundson (2015)	Canada	33	Community	36.9(+/−11.2)	Female-25(76)	White-26(79)	Not reported	Not reported	Not reported
Goldstein et al. (2018)	USA	47	Community care	47.4(+/−15.9)	Male-17(80.9)	White-12(57.1)	46.3(+/-14.4)	Male-21(80.8)	White-13(50.0)
Gelkopf et al. (2013)	Israel	42	Rehab unit (Army)	39.1(+/−12.4)	Male-22(100)	Not reported	37.5(13.6)	Male-20(100)	Not reported
Hall et al. (2020)	USA	54	Community care	67.7(+/−3.2)	Male-34(94)	African Caribbean-33(92)	66.9 (+/-4.3)	Male-15 (83)	African-Caribbean- 13(72)
Johnson et al. (2018)	USA	38	Community care	54.3(+/−12.8)	Male-32(84.2)	Not reported	Not reported	Not reported	Not reported
Kim et al. (2013)	USA	22	Secondary care	57.6 (+/−7.7)	Female– 10 (91)	White- 6(55)	45.0 (+/-10.0)	Female-11 (100)	White- 7(64)
Mitchell et al. (2014)	USA	38	Community care (Veteran medical center)	44.4(+/−12.4)	Female-20(52.6)	White−12(60)	Not reported	Female-18(47.4)	White-8(44.4)
Powers et al. (2015)	USA	9	Community (online advertisement)	34(+/−11.8)	Female-8(89.9)	White-8(88.9)	Not reported	Not reported	Not reported
Reinhardt et al. (2018)	USA	51	Community	44.1(+/−13.9)	Male-24(47.0)	White-16(31.4)	46.6(12.7)	Male-10(19.6)	White-9(17.6)
Rosenbaum et al. (2015a,b)	Australia	81	Inpatient unit	47.1 (+/−11.3)	Female- 3 (8)	Not reported	52.0 (+/-12.7)	Female-10 (24)	Not reported
van der Kolk et al. (2014)	USA	64	Community advertisement	41.5(+/−12.2)	Female- 32(100)	White-25(78.1)	44.3(+/-11.9)	Female-32(100)	White-25(78.1)
Whitworth et al. (2019a)	USA	30	Community	27.7(+/−5.9)	Female-11(73.3)	White-4(26.7)	30.5(+/-8.7)	Female-11(73.3)	White-7(46.7)
Whitworth et al. (2019b)	USA	22	Community	33.8(+/−11.1)	Female-9(81.8)	Black or African American-4(36.4)	32.1(+/-15.6)	Female-9(81.8)	Black or African American-4(36.4)

### Risk of bias assessment

Key features that impacted upon the methodological quality of each reviewed study are presented in Table 2. There is a significant risk of bias across the studies, with the overall risk of bias considered high or with some concerns. This was primarily the case with regard to allocation concealment, blinding of participants and key research team members, selective reporting, and blinding of outcome assessment. Seven studies (Gelkopf et al., 2013; Mitchell et al., 2014; van der Kolk et al., 2014; Powers et al., 2015; Johnson et al., 2018; Reinhardt et al., 2018; Whitworth et al., 2019a) were rated as having high risk of bias, five (Kim et al., 2013; Fetzner and Asmundson, 2015; Rosenbaum et al., 2015a,b; Goldstein et al., 2018; Whitworth et al., 2019b) with some concerns and only one (Hall et al., 2020) deemed low risk. Follow-up and attrition rates were poorly defined; most studies adequately described the follow-up period, and only one provided an adequate description of rates of and reasons for dropout (Hall et al., 2020). Only one study had a low risk of bias in all criteria of the checklist (Hall et al., 2020). For all studies, the analytical approach utilized was considered appropriate. Five studies (Kim et al., 2013; Fetzner and Asmundson, 2015; Powers et al., 2015; Whitworth et al., 2019a,b) reported a small sample size acknowledging this as a major limitation and demonstrating a paucity and poor quality of sample size calculations. Additionally, one study reported an imbalance in cases vs. control as a major limitation because of inadequate control counterpart (Powers et al., 2015).

**Table 2:** Risk of bias assessment.

**Study**	**Rosenbaum et al. (2015a,b)**	**Hall et al. (2020)**	**Goldstein et al. (2018)**	**van der Kolk et al. (2014)**	**Mitchell et al. (2014)**	**Reinhardt et al. (2018)**	**Whitworth et al. (2019a)**	**Whitworth et al. (2019b)**	**Powers et al. (2015)**	**Fetzner and Asmundson (2015)**	**Gelkopf et al. (2013)**	**Kim et al. (2013)**	**Johnson et al. (2018)**
Domain 1													
Domain 2													
Domain 3													
Domain 4													
Domain 5													
Overall RoB	Some concerns	Low risk	Some concern	High risk	High risk	High riak	High risk	Some concerns	High risk	Some concerns	High risk	Some concerns	High risk

Key Domain 1: Bias arising from the randomization process. Domain 2: Bias due to deviations from intended interventions (effect of allocation to intervention). Domain 3: Bias due to missing outcome data. Domain 4: Bias in measurement of the outcome. Domain 5: Bias in selection of the reported result. 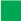 Low risk. 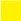 Some concerns. 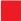 High risk.

### Certainty of evidence

For all 13 studies, the result of the GRADE was very low suggesting the true effect is likely to be substantially different from the estimated effect (Schünemann et al., 2013) (Table 3). Three studies (Kim et al., 2013; Rosenbaum et al., 2015a,b; Goldstein et al., 2018) used multiple types of exercise intervention and a GRADE ranking was undertaken. The GRADE ranking indicates low certainty that the exercise interventions are effective at reducing PTSD symptoms, with small RCTs indicating larger fully-powered trials are needed to adequately test these assumptions. Three studies (Mitchell et al., 2014; van der Kolk et al., 2014; Reinhardt et al., 2018) investigated the effect of yoga on PTSD symptoms; the GRADE ranking indicated moderate/low certainty that there is no effect on PTSD symptoms following a yoga intervention. Four studies (Powers et al., 2015; Goldstein et al., 2018; Whitworth et al., 2019a,b) investigated the effect of resistance training and aerobic exercise and PTSD symptoms; the GRADE ranking was low for both interventions, meaning that we have little certainty that the observed effects are the true effects of these interventions. Three further studies (Gelkopf et al., 2013; Kim et al., 2013; Johnson et al., 2018) tested the effectiveness of sailing, mindfulness-based stretching and deep breathing exercise, and therapeutic horseback riding; these were rated as either low or very low.

**Table 3:** GRADE rating on the level of evidence of the included studies.

	**Quality assessment**							**Number of patients**		**Quality**
**Intervention**	**No of studies**	**Design**	**Risk of bias**	**Inconsistency**	**Indirectness**	**Imprecision**	**Publication bias**	**Intervention**	**Comparison**	
Yoga	3	RCT	Serious limitations	No serious inconsistency	Serious indirectness	Serious imprecision	Detected	78	75	VERY LOW
Multi	3	RCT	Serious limitations	No serious inconsistency	Serious indirectness	Serious imprecision	Detected	96	88	VERY LOW
Resistance	2	RCT	Serious limitations	No serious inconsistency	No serious indirectness	Serious imprecision	Detected	26	26	VERY LOW
Aerobic	2	RCT	Serious limitations	No serious inconsistency	Serious indirectness	Serious imprecision	Detected	27	15	VERY LOW
THR	1	RCT	Serious limitations	None	Serious indirectness	Serious imprecision	Detected	15	14	VERY LOW
Sailing	1	RCT	Serious limitations	None	Serious indirectness	Serious imprecision	Detected	22	20	VERY LOW
MBX	1	RCT	Serious limitations	None	Serious indirectness	Serious imprecision	Detected	11	11	VERY LOW
**Outcome:**										
**PTSD scores**										

THR, Therapeutic Horseback Riding; PTSD, Post Traumatic Stress Disorder; MBX, Mindfulness-Based Exercise.

### Direction of effect

The direction of effect plot (Table 4) indicates a beneficial direction of effect for all exercise intervention except resistance that suggested no change. A sign test was not performed following the direction of effect plot, due to the small number of studies limiting its power and the detection of publication bias in the studies (Nikolakopoulos, 2020).

**Table 4:** Direction of effect Plot, RoB and certainty of evidence.

**Intervention**	**Study**	**Direction**	**GRADE**
		**of effect**	
**Multi**	Rosenbaum et al., 2015a,b	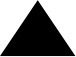	
	Hall et al., 2020	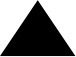	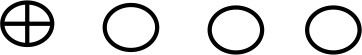
	Goldstein et al., 2018	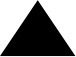	
**Yoga**	van der Kolk et al., 2014	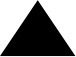	
	Reinhardt et al., 2018	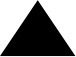	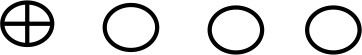
	Mitchell et al., 2014	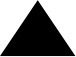	
**Resistance**	Whitworth et al. (2019a)	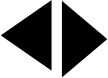	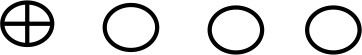
	Whitworth et al. (2019b)	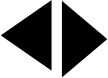	
**Aerobic**	Fetzner and Asmundson (2015)	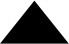	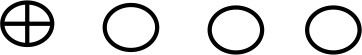
	Powers et al., 2015	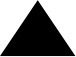	
**Sailing**	Gelkopf et al., 2013	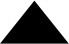	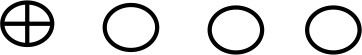
**MBX**	Kim et al., 2013	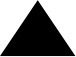	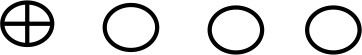
**THR**	Johnson et al., 2018	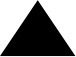	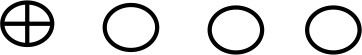

Key

Effect direction

Upward arrow ▴= positive health impact

Downward arrow ▾= negative health impact

Sideways arrows ◂▸= no change/mixed effects/conflicting findings

Sample size

Large arrow ▴ > 25 individuals in the intervention group

Medium arrow ▴ = 15 15 to 25 individuals in the intervention group

Small arrow ▴ < 15 individuals in the intervention group

Risk of bias

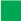
 = low risk of bias

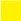
 = some concerns

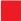
 = high risk of bias

Certainty of evidence (GRADE)

⊕⊕⊕⊕ = High

⊕⊕⊕○= Moderate

⊕⊕○○= Low

⊕○○○= Very low.

### Characteristics of included trials

#### Outcome measures characteristics

We noted significant diversity in the diagnostic criteria utilized to ascertain the presence of PTSD symptoms. The most important differences were: (1) where studies used a validated diagnostic tool or screening instrument; (2) Clinician rated assessment (3) Symptoms checklist. Five studies used the self-administered PTSD Checklist for Civilians (PCL-C) (Kim et al., 2013; Mitchell et al., 2014; Fetzner and Asmundson, 2015; Rosenbaum et al., 2015a,b; Reinhardt et al., 2018) and three studies used the Clinician-Administered PTSD Scale (CAPS) (van der Kolk et al., 2014; Goldstein et al., 2018; Reinhardt et al., 2018). Two studies used the PTSD Checklist for military personnel (PCL-M) (Johnson et al., 2018; Reinhardt et al., 2018) and two studies used the self-reported Post Traumatic Stress Diagnostic Scale (PDS5) (Whitworth et al., 2019a,b). One study used the Post-Traumatic stress Symptomology (PTS) (Gelkopf et al., 2013), two studies used the self-administered PTSD Checklist (PCL-5) (Whitworth et al., 2019a; Hall et al., 2020) and one study used the clinician-rated assessment of the PTSD Symptom Scale-Interview (PSSI) (Powers et al., 2015).

#### PTSD diagnosis at baseline

Five studies (Powers et al., 2015; Rosenbaum et al., 2015a,b; Goldstein et al., 2018; Johnson et al., 2018; Reinhardt et al., 2018) recruited participants with a DSM-IV diagnosis of PTSD, two (Johnson et al., 2018; Whitworth et al., 2019b) with DSM-V, and a further two studies (Kim et al., 2013; Fetzner and Asmundson, 2015) with PCL-C. One study recruited participants with probable PTSD based on the primary care screening from DSM-5 followed by a clinical interview (Mitchell et al., 2014). Another study used a modified 30-item Stanford Acute Stress Reaction Questionnaire (SASRQ) to establish a diagnosis of PTSD (Gelkopf et al., 2013). Table 5 provides detailed descriptions of the diagnostic criteria and PTSD measure used in the reviewed studies.

**Table 5:** Intervention and control component characteristics.

**Author (Year)**	**Number of trial arms**	**Baseline PTSD; reported measure**	**Intervention**	**Intervention duration**	**Frequency**	**Time**	**Control**	**Follow-up – post intervention**	**Outcome measure – pre and post intervention**	**Study results post-intervention Mean (SD), SMD (Cohen's *d*)**
Rosenbaum et al. (2015a,b)	2	DSM-IV-TR diagnosis of PTSD checklist – PCL-C	Resistance Exercise and a walking programme and exercise diary	12 weeks	x1wk Sup x2 wk HEP and daily walking 10,000 steps	30 mins	A combination of psychotherapy, pharmaceutical interventions, and group therapy facilitated by psychologists.	12 weeks	PTSD symptoms assessed via the PTSD checklist–civilian version (PCL-C)	PTSD symptoms in (intervention v/s control group) - mean di?erence = −5.4, 95% CI (−10.5 to−0.3), *p* = 0.04
Kim et al. (2013)	3	PTSD Checklist–Civilian version (PCL-C) scores of at least 28	Stretching and deep breathing exercise (MBX)	8 weeks	x2 weekly	60 mins	Control group	16 weeks	PTSD Checklist–Civilian version (PCL-C) scores of at least 28	PCL-C symptom scores – MBX v/s control- mean difference = −13.6; 95% confidence interval [CI], (−25.6 to −1.6), p= 0.01
Hall et al. (2020)	2	DSM-V diagnosis of PTSD checklist – PCL-5	Individualized exercise sessions	12 weeks	x3 weekly	60–90 mins	Care provided at women's health, mental health, or geriatrics clinics.	12 weeks	The PTSD checklist (PCL-5)	PCL-5 symptoms – Exercise v/s control – Mean difference=-4.23, 95% CI (−11.7 – 3.3); Cohen's *d* (0.38)
Whitworth et al. (2019a)	2	The PTSD checklist (PCL-5)	Resistance training	3 weeks	x3 weekly	30 mins	Nine 30-min sessions, involving videos on various educational topics excluding exercise and mental health	3 weeks	Posttraumatic Stress Diagnostic Scale for DSM-5 checklist – PCL-5	PCL5 symptoms scores – exercise v/s control = d = −0.87; 95% CI (0.03, 1.70)
Goldstein et al. (2018)	2	(DSM-IV; Criteria for current PTSD or partial PTSD. - CAPS	Aerobic, resistance training and yoga	12 weeks	x3 weekly	60 mins	Waitlist control group, receiving same intervention after 12 weeks	12 weeks	PTSD related symptoms- assessed via CAPS interview based on the DSM-IV	PTSD symptom severity v/s waitlist control - Mean = 14.77(SD +/−24.56); Cohen's d = −0.90 [95% CI: −1.72, −0.08]
Powers et al. (2015)	2	PTSD based on DSM-IV criteria- Clinical assessment - PSSI	Aerobic and exposure therapy session (PE+E)	12 weeks	x1 weekly	30 mins	Prolonged exposure therapy alone	12 weeks	Clinician-rated assessment (PTSD Symptom Scale-Interview; PSSI	PE + E v/s PE- pre- and post-PSSI Means scores = 42.00 and 5.20. Cohen's *d* = 2.67 (large effect size between group)
Fetzner and Asmundson (2015)	3	PTSD checklist – PLC-C	[1] Attention to somatic arousal and aerobic exercise [2] Distraction from somatic arousal and aerobic exercise	2 weeks	x3 per week 6 sessions-not more than 4	20 mins	Aerobic exercise with no distraction	4 weeks	PCL-C scores	Effect sizes for PTSD – CD/IP groups v/s aerobic only- CD group = (Cohen's *d* = 1.18, large effect size)
Whitworth et al. (2019b)	2	Posttraumatic Diagnostic Scale for DSM-5 (PDS5	High intensity resistance training	3 weeks	x3 per week	30 mins	30-min sessions, participants learned about various topics including nutrition, human anatomy, the universe) through videos and handouts	3 weeks	PDS-5 scores	PDS5 scores- Intervention v/s control = No observed group di?erences for total PTSD symptoms – variance between group F_(1,17)_ = 0.01, *p* = 0.91)
Johnson et al. (2018)	2	PTSD Checklist-Military Version (PCL-M). The PCL-M−17 DSM-IV symptoms of PTSD	Therapeutic horseback riding (THR)	6 weeks	x1 per week	40 mins	Waitlist control group	6 weeks	PCL-M scores	THR v/s waitlist control, PCL-5 symptoms = Week 3, F_(1,17)_ = 10.678, *p* = 0.005, Between week 3 and week = F_(1,17)_ = 8.750, *p* = 0.009.
Gelkopf et al. (2013)	2	PTS measured by a modified 30-item Stanford Acute Stress Reaction Questionnaire (SASRQ)	Nature adventure rehabilitation (NAR)	12 months	x1 per week	180 mins	Waitlist control	12 months	PTS symptoms scores	NAR v/s waitlist control at 1 year follow-up – *F* = 4.44; *p* = 0.04
van der Kolk et al. (2014)	2	Clinician administered PTSD scale using CAPS>45 scoring rules	Yoga	10 weeks	x1 per week	60 mins	Weekly women's health education	5 and 10 weeks	PTSD scale using CAPS>45 scoring rules scores	Yoga v/s control-Cohen's *d* = 1.07 (large effect size between group)
Mitchell et al. (2014)	2	Presence of at least one symptoms in each DSM criteria cluster or meeting criteria for at least 2 symptoms clusters	Yoga	12weeks or 6 week	1x per week or x2 per week	75 mins	Assessment control group	12 weeks	PSS-I scores	Yoga group mean PSS-I score = −0.10 (*p* = 0.003); Control group = −0.09 (*p* = 0.02); Between-groups effect sizes were small to moderate (0.08–0.31)
Reinhardt et al. (2018)	2	DSM-IV TR	Yoga	10 weeks	2x per week	90 mins	Assessment only control group	10 weeks	PCL-M and PCL-C scores	Yoga group CAPS mean (SD) baseline: 70.3 (SD+/−18.5), past week mean SD: 58.2 (SD+/−26.5), Past months baseline: 73.3 (SD+/– 18.3); Past months: 69.2(SD+/−25.7)

DSM, Diagnostic and Statistical Manual of Mental Disorder; PTSD, Post Traumatic Stress Disorder; MBX, Mindfulness Based Exercise; CAPS, Clinician Administered PTSD Scale; PTS, Post Traumatic Stress; THR, Therapeutic Horseback Riding; PDS, Post Traumatic Diagnostic Scale; NAR, Nature Adventure Rehabilitation; SASRQ, Stanford Acute Stress Reaction Questionnaire.

### Follow up assessment points

Five studies had follow-ups at 12 weeks following the end of the intervention period (Mitchell et al., 2014; Powers et al., 2015; Rosenbaum et al., 2015a,b; Goldstein et al., 2018; Hall et al., 2020). Of two studies with 10 weeks duration, one had a follow up at 10 weeks (Reinhardt et al., 2018) and the second at both 5 and 10 weeks (van der Kolk et al., 2014). One study had a follow-up point of 1 year following a 12-month intervention (Gelkopf et al., 2013). One study of 8 weeks duration had follow-ups at 4, 8 and 10 weeks (Kim et al., 2013). One study had a follow-up at the end of the 6-week intervention (Johnson et al., 2018). One study had a 4-week follow up assessment point after a 3-week intervention (Whitworth et al., 2019a). One study had a follow-up at the end of a 3-week intervention (Whitworth et al., 2019b). One study of 2 weeks duration had a follow-up directly after the intervention and additionally at 1 and 4 weeks (Fetzner and Asmundson, 2015).

### Effect of exercise/physical activity on PTSD symptoms and intervention characteristics

#### Multiple forms of exercise

Three studies (182 participants) combined multiple types of exercise in an intervention of 12 weeks duration (Rosenbaum et al., 2015a,b; Goldstein et al., 2018; Hall et al., 2020). One study combined aerobic, strength, balance and flexibility exercise, delivered over a span of 12 weeks with each session lasting for 60–90 min three times a week. This study reported a moderate between-group improvement in PTSD symptoms [standardized mean difference = −4.23, Cohen's *d* = de (95% CI −11.7 to 3.3)], participants in the intervention group showed a 16% improvement in PTSD symptoms whereas those in the waitlist group demonstrated a 7% improvement post-intervention (Hall et al., 2020). Another study that combined resistance exercise and walking for 30 min, three times a week over a 12 week period resulted in a significant between-group reduction in PTSD symptoms [mean difference = −5.4, 95% CI (−10.5 to −0.3), *p* = 0.04] (Rosenbaum et al., 2015a,b). One study which combined aerobic exercise, strength training with weights and resistance bands and yoga movements for 60 min per session three times a week over a 12 week period, reported a significant reduction in PTSD symptom severity compared to the waitlist control group [standardized mean difference = 14.77(SD +/−24.56); Cohen's *d* = −0.90 [95% CI: −1.72, to 0.08] (Goldstein et al., 2018). Two studies included military veterans (101 participants) (Goldstein et al., 2018; Hall et al., 2020) and one recruited adults inpatients within a psychiatric hospital (81 participants) (Rosenbaum et al., 2015a,b). Participants in all studies were predominantly male, with one study conducted on a much older age group (60–78 years) (Hall et al., 2020). All three studies used different PTSD symptom measures and all suggested a beneficial direction of effect for the PTSD scores; however, the certainty of evidence across the studies was very low.

#### Yoga

Three studies (153 participants) investigated yoga (Mitchell et al., 2014; van der Kolk et al., 2014; Reinhardt et al., 2018). Two studies reported basing the intervention on Kripalu yoga, which combines physical postures, breathing, and meditation (Mitchell et al., 2014; Reinhardt et al., 2018). Both studies reported a small between group reduction in PTSD symptoms [mean difference = 0.02 (+/4.7); Cohen's *d* = 0.02; variance F test = 3.41; effect size (0.04, small), *p* = 0.09]. The third study reported the intervention based on Hatha yoga, which also utilized breathing, postures, and meditation and reported a significant between group reduction in PTSD symptoms [yoga vs. control-Cohen's *d* = 1.07 (large effect size between group)] (van der Kolk et al., 2014). Two studies (van der Kolk et al., 2014; Reinhardt et al., 2018) were of 10-weeks duration for 60–90 min twice a week and the third study was of either 12 weeks or 6 weeks depending on whether the yoga session was performed once or twice per week for 75 min per session (Mitchell et al., 2014). Two studies included only females (102 participants) with a mix of 93 civilians and 9 veterans (Mitchell et al., 2014; van der Kolk et al., 2014), and the third study consisted mainly of males (45 males, 6 females) who were either serving military personnel or veterans (Reinhardt et al., 2018). Two studies used the CAPS measure (van der Kolk et al., 2014; Reinhardt et al., 2018), with one of these studies also using the PCL-C and PCL-M (Reinhardt et al., 2018), with the PCL-C also being shared with the third study (Mitchell et al., 2014). All three studies suggested a beneficial direction of effect, but the overall certainty of the evidence was very low.

#### Resistance training

Two studies (52 participants) examined the effectiveness of a resistance exercise intervention over a 3-week period for 30 min, three times a week in an adult population with PTSD, living in the community (Whitworth et al., 2019a,b). The resistance exercise intervention was not effective at reducing PTSD symptoms compared to the control group [(Cohen's *d* = −0.87; 95% CI (0.03, 1.70); *p* = 0.05)] (Whitworth et al., 2019a). Additionally, the other study observed no group difference in PTSD symptoms (F = 0.01, *p* = 0.91) (Whitworth et al., 2019b). Of the 52 participants across the two studies, 77% were female. Both studies consisted of five resistance exercises: squat, bench press, pulldown, overhead press and biceps curl. Both studies used the same outcome measure of PDSD but both studies suggested no overall change to PTSD outcome scores. The certainty of evidence was very low for this intervention.

#### Aerobic exercise

Two studies (42 participants) investigated the effectiveness of aerobic exercise on adults living in the community, diagnosed with PTSD (Fetzner and Asmundson, 2015; Powers et al., 2015). Both studies reported a significant between group difference in the reduction of PTSD following aerobic exercise mea*n* = 42.0 (Cohen's *d* = 2.67) and Cohen's *d* = 1.18 respectively. Across the two studies, 79% of the participants were female. One study was of 12 weeks duration (one session per week), lasting 30 min (Powers et al., 2015), while the second study was of 2 weeks duration but with sessions three times a week (i.e., total of 6 sessions), each session was 20 min duration (Fetzner and Asmundson, 2015). The two studies used different PTSD outcome measures but both studies suggested a beneficial direction of effect, although the certainty of the evidence was very low.

#### Sailing

One study (42 participants) trialed a weekly, 180 minutes per session sailing intervention over a one-year period with male military veterans (Gelkopf et al., 2013). This study reported a statistically significant reduction in PTSD following sailing compared to the control group (F: 4.44; *p* = 0.04). The PCL-M was used as the outcome measure as they were military. The results implied a beneficial direction of effect, but the study was at high risk of bias and the certainty of the evidence was very low.

#### Mindfulness-based stretching and deep breathing exercise

One study (29 participants) investigated a twice a week, 60 min per session for 8 weeks mindfulness-based exercise intervention with adult nurses (97% female) (Kim et al., 2013). This study reported a superior group reduction in PTSD following the mindfulness- based exercise intervention compared to the control group (mean difference: −13.6; 95% (CI) [−25.6 to −1.6], *p* = 0.01). The PCL-C PTSD outcome measure was used with the results suggesting a beneficial direction of effect. The study was judged to be of moderate risk of bias and the certainty of evidence was very low.

#### Therapeutic horseback riding

One study (29 participants) evaluated an intervention of therapeutic horseback riding on military veterans, once a week for 6 weeks, each session lasting for 40 min (Johnson et al., 2018). This study reported significant between group differences in PTSD symptoms in the horse-riding group compared to a control group; symptoms significantly decreased between baseline and week 3, (F = 10.7, *p* = 0.005), and between week 3 and week 6 of riding, (F = 8.750, *p* = 0.009). The study utilized the PCL-M measure of PTSD and the results suggested a beneficial direction of effect, but the study was at high risk of bias and the certainty of evidence was very low.

## Discussion

This systematic review is the first to investigate and compare different forms of exercise/physical activity for their ability to reduce symptoms of PTSD. The review suggested that there was a beneficial direction of effect for all exercise/physical activity interventions except resistance exercise. An explanation as to why no direction of effect in the two studies investigating resistance exercise (Whitworth et al., 2019a,b) might be due to the short follow-up period (3 weeks), which was one of the shortest compared to the other reviewed studies. None of the exercise/physical activity interventions had any detrimental effects on PTSD outcome scores. Overall, the results indicate that there is better quality of evidence for multimodal exercise interventions (a mixture of cardiovascular and resistance training) having a beneficial direction of effect on PTSD symptoms. However, the GRADE assessment indicates only moderate certainty of evidence that exercise interventions are effective at reducing PTSD symptom, primarily due to concerns about wide variation between the included studies.

The findings of this review suggests that overall the risk of bias was high in approximately 50% of the included studies. The number of randomized controlled trials for each exercise type was limited, with three types only evaluated within a single study (mindfulness stretching and breathing, sailing and horse riding). Wide heterogeneity was seen in the use of PTSD outcome measures across the studies despite gold standards for PTSD measurement being reported (Hall et al., 2020). With small sample sizes and lack of reliability, the evidence is not strong enough to draw firm conclusions and determine which exercise intervention is the most effective in reducing PTSD symptoms. However, even though the overall effect size could not be reported within a meta-analysis, this review shows that exercise does have a beneficial effect on PTSD symptoms.

This review adds to previous systematic reviews from Rosenbaum et al. (2015a,b) and McGranahan and O'Connor (2021), who concluded that exercise has a beneficial effect on PTSD symptoms. However, the current review has highlighted that other physical activities, such as sailing (Gelkopf et al., 2013), therapeutic horseback riding (Johnson et al., 2018), and mindfulness-based stretching and deep breathing exercise (Kim et al., 2013), may also be beneficial. These interventions require further investigation as studies were singular and had small sample sizes. Additionally, the current review adds to a previous systematic review by Whitworth and Ciccolo (2016), exploring the effect of exercise on PTSD symptoms in military veterans. Their review included two non-randomized trials compared to the thirteen randomized controlled trials included in the current review, of which five were on military populations (Gelkopf et al., 2013; Goldstein et al., 2018; Johnson et al., 2018; Reinhardt et al., 2018; Hall et al., 2020).

## Strengths and limitations

The current review has several strengths. The protocol of this systematic review was registered prospectively online (PROSPERO) and the review adheres to the PRISMA guidelines. All stages of the review were conducted by two reviewers, including the risk of bias assessment and GRADE evaluation. However, there are some limitations which should be noted. Significant heterogeneity was found between the included studies, particularly pertaining to methods of ascertaining PTSD and physical/exercise type which precluded meta-analysis. A direction of effect plot was used, but this only focused on whether there is evidence of an effect, rather than the average size of effect (Campbell et al., 2020). The gender of participants in individual studies were often imbalanced or of a single gender, although overall the studies were more balanced with 54% males and 46% females. The study populations were heterogeneous in terms of participants being civilian, military or inpatients, so the results do not represent any one population. Furthermore, the effect of the different frequency, length of session and intensity of exercise/physical activity on PTSD has not been fully investigated in this review, further studies would be useful to address this aspect.

The research also did not differentiate between participants with PTSD and those with complex PTSD; where complex PTSD includes people who have experienced repeated traumatic events either as a child or as an adult (Bisson et al., 2015). Therefore, exercise/physical activity interventions and their results could be different for these individuals. Publication bias could have also influenced the results of this review due to small sample sizes and associated reduced methodological quality compared to larger studies (Friedman, 2019). Additionally, there were limitations in the study selection process such as restricting the search to English language publications.

## Future research

Given that a number of different exercise and physical activities have been evaluated for reducing PTSD with quite different dynamics i.e., ranging from individual-based exercise to group activity and those such as sailing or horse-riding, it would be beneficial to understand which components of exercise are most important and how beneficial effects on PTSD are mediated (Light and Pillemer, 1984). Given that the best quality of evidence supported multimodal exercise intervention (i.e., combined cardiovascular and resistance training) these should be recommended for those with PTSD. Furthermore, future research should consider exploring whether there is an interaction between gender and the effects of exercises on PTSD symptoms.

## Conclusion

This review quantifies the effect of different exercise and physical activity interventions on PTSD symptoms and demonstrated that physical/exercise interventions shows promise to improve PTSD symptoms. Adequately powered additional RCTs are required to provide more definitive evidence of a causal relationship between different types of exercise/physical activity and PTSD. Significant heterogeneity was found between the included studies, particularly pertaining to diagnosing PTSD, sample size, population and method employed, which precluded meta-analysis. Nonetheless, this review indicated that exercise and physical activity shows potential for improving PTSD symptoms.

## Data availability statement

The original contributions presented in the study are included in the article/Supplementary material, further inquiries can be directed to the corresponding author/s.

## Author contributions

NL, NM, and DF contributed equally to the conception of this review. NL and NM performed screening for study selection, collected data from the included studies, and conducted quality assessment. FJ drafted the first version of this review and this was reviewed/revised by DF and DE. The final version was drafted by FJ. The search strategy was developed by NL and iteration discussed with NM and DF. The final version was approved by DF and DE. The search was performed by NL. FJ and NL performed data analysis/synthesis. NM ensured data extraction consistency. FJ drafted the manuscript and all authors critically reviewed the manuscript. All authors contributed to the article and approved the submitted version.

## Conflict of interest

The authors declare that the research was conducted in the absence of any commercial or financial relationships that could be construed as a potential conflict of interest.

## Publisher's note

All claims expressed in this article are solely those of the authors and do not necessarily represent those of their affiliated organizations, or those of the publisher, the editors and the reviewers. Any product that may be evaluated in this article, or claim that may be made by its manufacturer, is not guaranteed or endorsed by the publisher.
