# Supplementary material for: Is exercise/physical activity effective at reducing symptoms of post-traumatic stress disorder in adults — A systematic review
Source: Front Psychol. 2022 Aug 12;13:943479. doi: 10.3389/fpsyg.2022.943479 (PMC9412746; doi:10.3389/fpsyg.2022.943479)
Supplement: Supplementary file 1 [file Data_Sheet_1.PDF]

Supplementary Material 1: Medline search strategy

1	exercis*.mp
2	movement therapy.mp
3	exercise training.mp
4	physical activit*.mp
5	aerobic exercise.mp
6	physical exercise.mp
7	resistance training.mp
8	strength training.mp
9	endurance exercise.mp
10	cardiovascular exercise.mp
11	CV.mp
12	stretch*.mp
13	sport*.mp
14	PTSD.mp
15	exp. post traumatic stress
16	post traumatic stress disorder.mp
17	posttraumatic stress.mp
18	1-13 OR
19	14-17 OR
20	18-19 AND
